# The Impact of Rural Population Mobility on Fertility Intention under the Comprehensive Two-Child Policy: Evidence from Rural China

**DOI:** 10.3390/ijerph19127365

**Published:** 2022-06-16

**Authors:** Qiang He, Xin Deng, Chuan Li, Zhongcheng Yan, Yanbin Qi

**Affiliations:** College of Economics, Sichuan Agricultural University, Chengdu 611130, China; heqiangklcf@stu.sicau.edu.cn (Q.H.); dengxin@sicau.edu.cn (X.D.); 2020108003@stu.sicau.edu.cn (C.L.); 2019108007@stu.sicau.edu.cn (Z.Y.)

**Keywords:** RPM, fertility intention, ESR model

## Abstract

Declining fertility rates pose challenges to global economic, social, cultural and political development. Low fertility rates among rural floating populations are exacerbating these challenges. However, it is not clear whether and to what extent rural population mobility (RPM) has reduced migrants’ willingness to have children. At the same time, rural migration may represent a self-selection behavior (i.e., selection bias), and traditional measurement methods may be insufficient for effectively estimating the quantitative impacts of rural migration. Accordingly, the data from 1734 rural households from 28 provinces in mainland China were collected in the current study, and endogenous switching regression (ESR) models were used to correct the selection bias to quantitatively evaluate the impacts of RPM on fertility intention. The results revealed the following: (1) For rural residents who choose to move, if they chose not to move, their willingness to give birth would increase by 19.820%, their willingness to have female children would increase by 48.526%, and their willingness to have male children would drop by 26.711%. (2) For rural residents who choose not to move, if they chose to move, their willingness to give birth would drop by 55.982%, their willingness to have female children would drop by 18.294%, and their willingness to have male children would drop by 55.106%. (3) For eastern rural residents who choose to move, if they chose not to move, their willingness to give birth would decrease by 40.273%. For midwestern rural residents who choose to move, if they chose not to move, their willingness to give birth would increase by 24.786%. (4) For eastern rural residents who choose not to move, if they chose to move, their willingness to give birth would increase by 11.032%. (5) For midwestern rural residents who choose not to move, if they chose to move, their willingness to give birth would drop by 71.744%. The abovementioned findings can provide research support for other low-fertility countries or regions toward increasing fertility rates and addressing any imbalances in current gender ratios. They can also help to provide realistic strategies for alleviating the global population crisis.

## 1. Introduction

Declining fertility rates pose economic, social, cultural and political challenges worldwide. Vollset et al. [1] predicted that the world’s population would reach a peak of approximately 9.7 billion in 2064 and then decline to approximately 8.8 billion in 2100. Additionally, it is believed that Asia and Central and Eastern Europe will experience the fastest declines in population, with the populations of China, Japan and South Korea (among 23 other countries and regions) decreasing by half. For example, Bumpass et al. [2] highlighted that low fertility rates led to Japan’s labor shortage, which could jeopardize the economic lifeline of the entire country. Additionally, Lee and Choi [3] predicted that fertility declines would cause an increase in the average age of Korean workers, from 39.1 years in 2010 to 43.0 years in 2050. Finally, Puig-Barrachina et al. [4] explained that low fertility rates have exacerbated the negative impacts on Spain’s welfare system stemming from the economic recession. Therefore, analyzing the fertility decline and identifying the precise countermeasures to inhibit such decline remain important.

**Figure 1:**
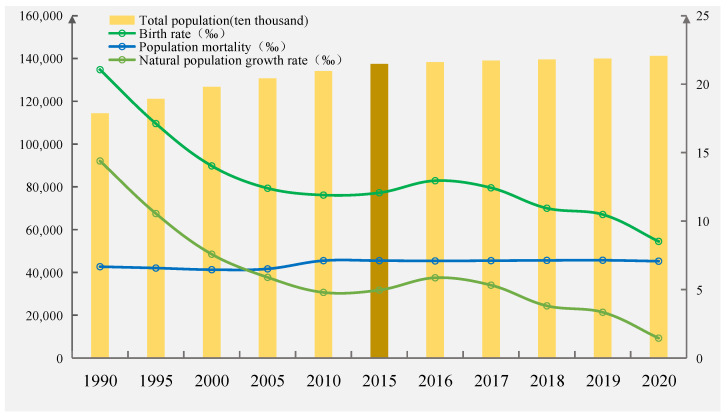
China’s birth rates, death rates, natural growth rates and total population.

China has become one country with a low fertility rate, and it is among the lowest in the world. According to the data collected through the seventh Chinese Census, China’s total fertility rate in 2020 was only 1.3, already below the warning line of 1.5. In 2015, the Chinese government introduced a universal two-child policy to avoid falling into the “low fertility trap” and to decelerate the growth of the country’s aging population and the reality of fewer children. In the short term, however, the universal two-child policy cannot effectively alleviate the abovementioned dilemma. For example, Zeng and Hesketh [5] explained that China’s universal two-child policy may help to deal with the challenges associated with an aging population, but due to the culture of low fertility introduced by previous policies, the implementation of the two-child policy is not likely to lead to a baby boom, only a moderate increase in fertility rates. In [6], the sample survey data of 1% of the Chinese population were analyzed, revealing that the universal two-child policy will not meet the expectation of a significant increase in birth rate. Similarly, Liu [7] found that any increase in the fertility rate brought on by the universal two-child policy was not obvious. Indeed, according to CNSB, there were only 950,000 more births in China in 2016 compared with 2015. As Figure 1 shows, between 1990 and 2020, China’s total population increased from 1.143 billion to 1.412 billion, the birth rate dropped from 21.06 per thousand to 8.52 per thousand, the death rate rose from 6.67 per thousand to 7.07 per thousand and the natural population growth rate dropped from 14.39 per thousand to 1.45 per thousand. Further, Figure 1 also shows that the natural growth and birth rates of China’s population in 2016 were higher than those for 2015, where a rare upward trend for the first time in 20 years can be seen. However, the trend of decline in the natural growth and birth rates has not changed, and the downward trend is showing annual increases. Therefore, exploring the reasons for fertility decline in China and the world is important.

In August 2021, the Chinese government decided to amend the Population and Family Planning Law to further improve the willingness of childbearing-age individuals to have children. Under the revised Population and Family Planning Law, a couple can have three children. The implementation of China’s three-child policy is conducive to improving the population’s age structure, reducing the dependency ratio of the elderly, increasing overall societal vitality and reducing the peak level of aging. However, we can also see objectively that factors such as high housing prices, high child-rearing costs and imperfect maternity services are inhibiting the fertility willingness of people of childbearing age, especially those in low-income groups. For example, Liu et al. [8] and Clark et al. [9] highlight that high housing prices significantly reduce people’s willingness to bear children, especially for families without houses. Similarly, Zeng and Hesketh [5] found that the opportunity and learning costs associated with education have a significant impact on parents’ fertility intention. On the other hand, Boldrin et al. [10] and Wang [11] revealed that perfect medical insurance and endowment insurance can improve people’s fertility intention. Additionally, the government’s level of education service [12] and the country’s ecological environmental pollution [13] and socially competitive environment [14] all have a direct or indirect impact on the fertility intention of childbearing-age individuals.

China is one of the largest developing countries in the world [15], but the fertility willingness among China’s rural floating population has not received much attention. Due to the low education of this population, individuals are often at a disadvantage in terms of employment competition, and most of them only find work in low-end positions in the secondary labor market [16,17,18]. The rural floating population is a unique group prone to risks associated with high unemployment [19] and health [20], as well as lack of corresponding social security [21]. China’s rural floating population is predominantly young and middle-aged, where individuals leave their homes for livelihood and their relationships, particularly their marriage relationships, are unstable [22]. With notions that a stable marriage is key to fertility-rate increases, the instability of this relationship poses a significant challenge [23]. However, the rural floating population can adopt measures to avoid these risks [24], particularly as such risks can leave individuals feeling anxious and depressed. Such flow-on effects may further impede individuals’ desire to procreate. Therefore, the current study investigated China’s unique rural floating population to determine their fertility intention, which may provide insights for China and other countries with low fertility rates into formulating important policies and strategies.

## 2. Literature Review

Since China’s reform and opening up, the country’s rural floating population is characteristically large, has a concentrated flow, and is young and middle-aged. The main reasons China’s rural population chooses to migrate are to work (or find work) and to do business. However, the migration of this population is fraught with risk and involves an uncertain future. Compared with local residents, migrants face many challenges in life and work [25]. Further, due to China’s long-standing urban-rural dual structure and household registration barriers, social security and social welfare systems are linked to the household registration system, making it difficult for the rural floating population to enjoy the same social security and welfare services accessible to local residents [26]. Therefore, uncertainty, a lack of security and perceived (and real) risk may represent the main factors causing a dilemma among this unique population.

Occupational risk is the main risk China’s rural floating population faces [27,28]. In terms of health risk, Wang et al. [29] found that migrant workers face higher occupational risk compared with local workers in China. Mucci et al. [30] conducted a literature analysis and determined that the main risks facing migrant workers include the development of infectious and metabolic cardiovascular diseases. Along with physical ailments, mental health problems are among the main risks faced by migrant workers [31,32]. For example, Ang et al. [33] found that Bangladeshi workers working in the Philippines are at higher risk of psychological distress than local workers. Zhu et al. [34] found that compared with other residents in Hunan Province, migrant workers appeared to be at a greater risk of hospitalization due to schizophrenia. Additionally, Li et al. [35] found that compared with other residents of Shanghai, migrant workers from other cities were more likely to experience depression. Migrant workers are also affected by uncertain risks. For example, Longling et al. [36] highlighted that uncertainty around income and expenditures significantly inhibited migrant workers’ living consumption. In recent years, the Chinese government has attached great importance to the social security of migrant workers. Unfortunately, however, issues such as unreasonable compensation for occupational diseases [37], household registration discrimination [38] and cultural integration pressure [39] cannot all be addressed at once. Although migrant workers do not represent the entirety of the rural floating population, they are a group that appear to best represent the hardships and risks faced by this population. Therefore, what is the fertility willingness among those in China’s rural floating population? Is there a difference in the desire of individuals in this population to have girls or boys? In China’s current climate, these are worthwhile questions to ask.

From the perspective of the impact of rural population mobility (RPM) on fertility intention, RPM is likely to largely affect fertility intention and fertility decision-making in the abovementioned population. For example, Bao-shu [40] found that the fertility rate among individuals in the rural floating population is not only lower than that of rural residents but also lower than that of urban residents. Farid et al. [41] found that the fertility attitudes and parenting behaviors among migrant workers were significantly different from those of traditional farmers. Additionally, there are several internal differences in the willingness of those in the rural floating population to have a second child. For example, Zhou and Guo [12] highlighted that if the first child of those in the rural floating population is a girl and they also live in underdeveloped cities, they are more willing to have a second child. The authors of [42,43] compared the willingness of local women and migrant women to have a second child and found that migrant women’s willingness was significantly lower. Of course, in addition to internal differences, external environmental factors can also affect the fertility willingness of those in the rural floating population. For example, Guo et al. [44] found that PM2.5 concentration significantly negatively impacted the floating population’s willingness to have a second child. Additionally, education resources [45] as well as accumulated mobile time [46] and medical insurance [47] also significantly impact the rural floating population’s willingness to have a second child. Based on a review of the literature on the rural floating population and their fertility intention, most studies have combined investigating the rural floating population and fertility intention, and thus, investigation directly into the negative impact of the rural floating population on fertility intention is scarce.

## 3. Data, Variables, and Method

### 3.1. Data

Data from the Chinese General Social Survey (CGSS) conducted by the China Survey and Data Center of Renmin University of China were used in this study. Founded in 2003, the CGSS is the earliest nationwide, comprehensive and continuous academic investigation program in China. The CGSS comprehensively collects data on social, community, family and individual factors, summarizes social-change trends in China, explores the important scientific and practical significance of topics, promotes the opening and sharing of scientific research, provides data for international comparative studies and acts as a multidisciplinary data acquisition platform to be used for society and the economy. Currently, the CGSS has become the most important data source for studies on Chinese society and is widely used in scientific research, as well as in teaching and government decision-making. In 2017, a total of 12,582 valid samples were collected by CGSS, including 783 variables. In the current study, cross-sectional data from the CGSS in 2017 were used. Based on the needs of the study, after deleting invalid cases, agricultural household registration samples and people of reproductive age (between 17 and 45 years old) were recruited. After handling the missing data and any outliers present, 1734 valid cases were finally selected.

### 3.2. Variables

#### 3.2.1. Dependent Variable

In the current study, rural residents’ fertility intention served as the dependent variable. To investigate reproductive intention, most studies in the extant literature examine childbearing-age individuals’ plan to have one child in a given period in the future [48,49]. However, based on China’s current fertility rates, this measure may not capture the final number of children born to people of childbearing age in the future. Therefore, the authors of the current study referred to studies by Liu and Gong [50], Meng and Lyu [51] and decided to use in the study’s questionnaire the following questions: How many children would you have if there were no policy restrictions? How many daughters would you have? and How many sons would you have? These questions were established as continuous variables.

#### 3.2.2. Key Variable

The study adopted rural population flow as the core independent variable. Most existing studies define the migrant population and migrant worker population as comprising those individuals who have been living for more than a given period as part of the floating population [52,53,54]. Considering that the current study investigated the quantitative relationship between rural population mobility and fertility intention, the question “Where is the location of your current household registration?” posed by Chen [55] was used to determine whether population movement had occurred. If the interviewee answered “Hometown (town, street)” and the location was the same as that of his/her household registration, this indicated the sample had not flowed, and thus, the value was 0. Accordingly, if a respondent answered, “Other townships (towns, streets) in this county (city, district)” and “Outside this city (district, county)”, the location of the sample was seen as inconsistent with the location of its registered residence, indicating that the sample had flowed, and thus, the value was 1. This index gave a binary variable of 0 or 1.

#### 3.2.3. Control Variables

This study referred to studies in the extant literature [56,57,58,59,60] to determine the control variables to be used, 12 in total. These were gender, age, education, nationality, belief, marital status, political parties, health, pension, fixed assets, family income and number of children. Additionally, the question “Is it difficult for rural people to obtain urban household registration? was used as an instrumental variable. If the interviewee chose comparative or complete agreement, the value of 1 was assigned, and the remainder of the answers were assigned 0. On the one hand, if respondents believed it was difficult for rural people to obtain urban household registration, their mobility enthusiasm decreased, most farmers’ enthusiasm gradually weakened, and it rarely grew stronger. On the other hand, in theory, this concept is not likely to directly affect farmers’ fertility. Therefore, the selected instrumental variables met the correlation conditions around the endogenous variables for this study. The model variables and summary statistics are described in Table 1.

### 3.3. Method

#### 3.3.1. Model Setting

According to the stochastic utility decision model proposed by Ali and Abdulai [61] and Becerril and Abdulai [62], whether farmers choose flow depends on the difference between the utility (*U*_1*i*_) of flow and the utility (*U*_0*i*_) of no flow. If $A_{i}^{*}=U_{1i}-U_{0i}>0$ farmers choose flow. In this study, the flow decision-making equation of farmers was defined as:

(1) $$A_{i}^{*}=\Phi \left(Z_{i}\right)+\mu_{i},\;\mathrm{if}\;A_{i}^{*}>0,\;\mathrm{then}\;A_{i}=1;\;\mathrm{otherwise}\;A_{i}=0$$

In Formula (1), $A_{i}^{*}$ is the latent variable; $A_{i}=1$ represents farmer *i* who chooses flow; $A_{i}=0$ represents farmer *i* who does not choose flow; and *Z_i_* is the vector of the exogenous explanatory variables, including individual variables and family variables that affect interviewees, as shown in Table 1. Finally, $\mu_{i}$ is the random disturbance term.

To measure the impact of rural population mobility on fertility intention, the current study developed the model of farmers’ fertility intention as follows:

(2) $$Y_{i}=X_{i}\beta_{i}+\delta A_{i}+\varepsilon_{i}$$

In Formula (2), the dependent variable *Y_i_* represents the fertility intention of farmers; *X_i_* represents the control variable vector; *A_i_* represents the flow variable of farmer *i*; and $\varepsilon_{i}$ represents the random disturbance term. Farmers choose to exhibit flow or not according to their conditions, the flow selection decision (*A_i_*) can be affected by some unobservable factors, and these factors can be related to the result variable (*Y_i_*), leading to a correlation between *A_i_* and $\varepsilon_{i}$ in Formula (2). Therefore, the direct estimation in Equation (2) may lead to estimation bias due to issues with sample self-selection. Referring to studies by Ma and Abdulai [63], Deng, Xu, Zeng and Qi [15], Deng et al. [64] and Deng et al. [65], this study selected the ESR model to address the sample self-selection problem.

The fertility intention models of farmers who choose to move and those who choose not to move can be represented as follows:

(3a) $$Y_{ia}=X_{ia}\beta_{a}+\sigma_{\mu a}\lambda_{ia}+\varepsilon_{ia},\;if\;A_{i}=1$$

(3b) $$Y_{in}=X_{in}\beta_{n}+\sigma_{\mu n}\lambda_{in}+\varepsilon_{in},\;if\;A_{i}=0$$

In Equations (3a) and (3b), *Y_ia_* and *Y_in_* represent the fertility intention of farmers who choose to move and those who choose not to move, respectively. *X_ia_* and *Y_in_* represent the factors influencing the fertility intention of the two types of farmers, as shown in Table 1. Both $\varepsilon_{ia}$ and $\varepsilon_{in}$ represent random perturbation terms. To address the problem of sample selection bias caused by unobservable factors, the inverse Mills ratios $\lambda_{ia}$ and $\lambda_{in}$, and the covariances $\sigma_{\mu a}=\mathrm{cov}\left(\mu_{i},\varepsilon_{ia}\right)$ and $\sigma_{\mu n}=\mathrm{cov}\left(\mu_{i},\varepsilon_{in}\right)$ were introduced in the current study. Further, the complete information maximum likelihood method was used to simultaneously estimate Equations (1) and (3a,b).

#### 3.3.2. Treatment Effect Estimation Method

By comparing the expected fertility intention of farmers who choose to move and those who choose not to move in a real and a counterfactual hypothetical scenario, the average processing effect of farmers choosing to move can be estimated as the expected value of the fertility intention of floating farmers:

(4) $$E\left[Y_{ia}|A_{i}=1\right]=X_{ia}\beta_{a}+\sigma_{\mu a}\lambda_{ia}$$

The expected fertility intention of farmers who choose not to move is given as follows:

(5) $$E\left[Y_{in}|A_{i}=0\right]=X_{in}\beta_{n}+\sigma_{\mu n}\lambda_{in}$$

At the same time, we can consider two counterfactual hypothesis scenarios, namely, the expected fertility intention of the floating farmers in the unused situation:

(6) $$E\left[Y_{in}|A_{i}=1\right]=X_{ia}\beta_{n}+\sigma_{\mu n}\lambda_{ia}$$

and the expected value of fertility intention of farmers who choose not to move under the condition of choosing to move:

(7) $$E\left[Y_{ia}|A_{i}=0\right]=X_{in}\beta_{a}+\sigma_{\mu a}\lambda_{in}$$

According to Equations (4) and (6), the processing effect of the fertility intention of farmers who choose to migrate can be given as follows:

(8) $$ATT_{i}=E\left[Y_{ia}|A_{i}=1\right]-E\left[Y_{ia}|A_{i}=1\right]=X_{ia}\left(\beta_{\alpha }-\beta_{n}\right)+\left(\sigma_{\mu a}-\sigma_{\mu n}\right)\lambda_{ia}$$

Similarly, the treatment effect of farmers who choose not to move can be given as follows:

(9) $$ATU_{i}=E\left[Y_{ia}|A_{i}=0\right]-E\left[Y_{in}|A_{i}=0\right]=X_{in}\left(\beta_{\alpha }-\beta_{n}\right)+\left(\sigma_{\mu a}-\sigma_{\mu n}\right)\lambda_{in}$$

In the current study, the average values of *ATT_i_* and *ATU_i_* were used to evaluate the average processing effect of two types of farmers choosing whether to move or not on fertility intention.

## 4. Results

### 4.1. Descriptive Results

#### 4.1.1. Collinearity Test

Before empirical analysis, a collinearity test on independent variables was conducted. The test results are given in Table 2. The VIF values for all variables were within 2, indicating no serious collinearity problem among the variables.

#### 4.1.2. Mean Differences

The mean difference helped to analyze the differences between farmers who choose to move and those who do not. The test results for the mean differences are shown in Table 3. All variables except DHG, gender and political party were significant at the 5% level, indicating significant differences between farmers who choose to move and those who do not. Among them, the mean difference of FI was −0.073, which was significant at the level of 5%, indicating the lower fertility willingness among farmers who choose to move than among of those who do not. Although the values in Table 3 reflect significant differences in the mean values of some variables in the case of whether farmers choose to move or not, it does not indicate that these differences are caused by rural population flow. To accurately demonstrate rural migrants’ fertility intention, the selection bias caused by the self-selection of the study sample was fully considered. Therefore, this study adopted a more scientific endogenous transformation model (ESR) to conduct empirical research.

### 4.2. Empirical Results

#### 4.2.1. The Determinants of RPM and FFI

Table 4 presents the estimation results of the models for farmers’ choice and their fertility intention. The two-stage equation independence LR test generated a significant result, and thus, the null hypothesis that the choice equation and the result equation are independent of each other at the 1% level was rejected. The Wald test of goodness of fit for simulation was significant at the 1% level. The correlation coefficient r1 of the error term was significantly positive at the 1% level, indicating a selection bias in the fertility intention model.

The ‘Selection’ column in Table 4 presents the determinants of the farmer mobility estimations based on the ESR model. The coefficient of education was found to be significantly positive (at the 1% level). The coefficients of the variables political parties (at the 5% level), pension (at the 1% level) and fixed assets (at the 1% level) were significantly negative, indicating that being a member of the Communist Party, participating in endowment insurance and having more houses significantly reduced farmers’ mobility. Additionally, the estimations for several variables produced some interesting results. For example, the coefficient for Lnincome was significantly negative (at the level of 1%), and the coefficient of Lnincome^2^ was significantly positive (at the level of 1%), indicating that farmers’ household income had a positive U-shaped relationship with choice flow. Further, the coefficient of the instrumental variables was significantly negative (at the 1% level), indicating that farmers’ perception of difficulty with regard to settling down in cities reduces their mobility.

The ‘Population mobility’ column in Table 4 represents the determinants of the FFI selection for the migrant farmers based on the ESR model estimates. The variables gender, political party, pension, fixed assets and Lnincome were found to significantly negatively affect farmers’ FFI, whereas the variables education, Lnincome^2^ and Num-children significantly positively affected farmers’ FFI. The ‘Population immobility’ column in Table 4 represents the determinants of FFI selection for non-mobile farmers based on the ESR model estimates. Gender, marriage and pension significantly negatively affected farmers’ FFI, while belief and Num-children significantly positively affected farmers’ FFI. In summary, this paper finds that in addition to the variables Num-children and gender, the variables education, party, pension, fixed assets, Lnincome, and Lnincome^2^ are not only the determinants of the selection of migrant farmers’ FFI but also the determinants of RPM. Most of these influencing factors reflect the income levels and family wealth of farmers. This shows that farmers’ incomes and family financial status are both determinants of the RPM and FFI of migrant farmers. In total, there were several differences in FFI determinants between farmers who do not choose to be mobile and those who do, indicating a difference between the two groups of farmers and also indicating that traditional measurement methods may not be sufficient for effectively estimating FFI. Further, the estimated results provided in Table 4 do not directly capture the quantitative impacts of farmer flow on FFI; therefore, a counterfactual framework needed to be developed to evaluate the quantitative impacts of farmer flow on FFI.

#### 4.2.2. Estimating the ATT and ATU of FFI

Table 5 presents the estimation results for farmers’ FFI impacted by the construction of a counterfactual framework based on the ESR model. First, for flowing farmers, Factual FFI was the FFI predicted by the ESR model (i.e., Factual FFI with a value of 1.887). Counterfactual FFI was based on the ESR model that predicted FFI if farmers who choose flow did not choose flow (i.e., Counterfactual FFI with a value of 2.261). Second, Factual FFI was the FFI predicted by the ESR model for farmers who do not choose flow (i.e., Factual FFI with a value of 1.956). Counterfactual FFI was based on the ESR model that predicts that farmers who do not choose flow have the FFI if they choose flow (i.e., the Counterfactual FFI was 0.861). The ATT value was determined to be −0.374, and its *t*-value was −26.256 (significant at the 1% level). This indicates that if farmers who choose to move do not choose to move, their FFI may increase by 19.820%. The value of ATU was −1.095, and its *t*-value was −96.633 (significant at the 1% level). This indicates that if farmers who do not choose flow choose flow, their FFI may decrease by 55.982%. Therefore, it can be said that RPM reduces farmers’ willingness to bear children.

#### 4.2.3. The Determinants of RPM and DHG/DHB

Table 6 presents the estimation results for the farmers’ choice flow model and the model for their willingness to bear female children. In the ‘Selection’ column, it can be seen that the coefficients of the variables Education and Lnincome^2^ were significantly positive, whereas the coefficients of the variables political parties, pension, fixed assets, Lnincome and IV were significantly negative. In the ‘Population mobility’ column, the coefficients of education, belief and Num-children were significantly positive. In the ‘Population immobility’ column, the coefficients of the variables education, Lnincome^2^ and Num-children were significantly positive, while the coefficients of the variables pension and Lnincome were significantly negative.

Table 7 gives the estimation results for the farmers’ choice flow model and the results of the model for willingness to bear male children. In the ‘Selection’ column, it can be seen the coefficients of the variables education and Lnincome^2^ were significantly positive, and the coefficients of the variables political parties, pension, fixed assets, Lnincome and IV were significantly negative. In the ‘Population mobility’ column, the coefficients of education, Lnincome^2^ and Num-children were significantly positive, and the coefficients of gender, party, pension, fixed assets and Lnincome were significantly negative. Additionally, in the ‘Population immobility’ column, the coefficients of belief and Num-children were found to be significantly positive, whereas the coefficients of gender, education, marriage and health were significantly negative.

#### 4.2.4. Estimating the ATT and ATU of DHG/DHB

Table 8 presents the estimation results for the counterfactual framework based on the ESR model for a discussion of the impacts of farmers’ mobility on their DHGS and DHBS. First, for DHG, the ATT value was −0.462, and its *t*-value was −52.559 (significant at the 1% level). This indicates that if farmers who choose to move do not choose to move, their DHG may increase by 48.526%. The value of ATU was −0.178, and its *t*-value was −26.020 (significant at the 1% level). This indicates that if the farmers who did not choose flow choose flow, their DHG may decrease by 18.294%. Second, for DHB, the ATT value was found to be −0.216, and its *t*-value was −30.965 (significant at the 1% level). This indicates that if farmers who choose to move do not choose to move, their DHB may decrease by 26.711%. The value of ATU was found to be −0.545, and its *t*-value was −1.0 × 10^2^ (significant at the 1% level). This indicates that if the farmers who did not choose flow choose flow, their DHB may decrease by 55.106%. Based on the comparison presented above, it can be argued that rural population migration has a greater impact on farmers’ willingness to bear male children than female children.

#### 4.2.5. Empirical Results at the Regional Level

Due to the vast territory of China, there are great differences in the development of rural areas in different regions. In order to compare whether there are differences in the impacts of rural population mobility on fertility willingness among different regions, this paper divides the sample into east and midwest for empirical analysis.

By comparing the empirical results in Table 9, we can see that for the east, the ATT value was 0.765, and its *t*-value was 34.594 (significant at the 1% level). This indicates that if farmers in the east who choose to move do not choose to move, their FFI may decrease by 40.273%. The value of ATU was 0.218, and its *t*-value was 8.781 (significant at the 1% level). This indicates that if the farmers in the east who do not choose flow choose flow, their FFI may increase by 11.032%. For the midwest, the ATT value was found to be−0.463, and its *t*-value was −19.488 (significant at the 1% level). This indicates that if farmers who choose to move do not choose to move, their FFI may increase by 24.786%. The value of ATU was found to be −1.400, and its *t*-value was −93.554 (significant at the 1% level). This indicates that if the farmers who do not choose flow choose flow, their FFI may decrease by 71.744%. The reason for this may be that the eastern region has higher economic and social development, and farmers have a more modern concept of fertility.

## 5. Discussion

Using data from the Rural General Social Survey, which covers 28 provinces in mainland China, the current study examined the impacts of rural migration on fertility intentions. Compared with previous studies, the marginal contributions of this study are as follows: (1) The study predominantly focused on the fertility intention of rural residents and discussed the theoretical mechanism of RPM on their fertility intention; (2) the study adopted the ESR model to correct the selection bias caused by observable and unobservable factors, as well as to evaluate the quantitative impact of RPM on their fertility intention; (3) through a comparative evaluation of the quantitative impact of RPM on farmers’ willingness to bear female or male children, the study finds that the impact of rural population mobility was greater for their willingness to bear male than for female children. The results of this study can support research conducted in other low-fertility countries or regions to improve fertility rates and address any imbalances in gender ratios. The results also point to practical strategies that can be implemented to alleviate the global population crisis.

The axiom “displaced from place to place” is truly representative of the floating population. This is especially true for rural migrants, who leave their homes to feed their families. At the same time, due to their low cultural literacy, most can only be engaged to work in labor-intensive industries, and they exhibit more pessimistic and helpless attitudes towards childbearing compared with others not living under the same conditions [66]. The findings of this study provide new evidence for this perspective. In recent years, however, the Chinese government has introduced several policies to increase the fertility rate toward alleviating problems associated with an increasingly aged population and reductions in the numbers of children being born (e.g., in 2013, the Chinese government issued the “only two children” policy, and in 2015, they issued the “universal two-child” policy). However, due to a lack of social security services for the rural floating population and their living environments, individuals in this population are unwilling to respond to the latter policy, rendering ineffective many of the policies and measures proposed by the Chinese government to increase fertility rates among this unique group. In addition, these people who choose to migrate tend to obtain more employment opportunities in cities than those who stay in rural areas. Most of them work hard. These will crowd out their personal living space and time, thereby reducing their willingness to have children. For example, Brinton et al. [67] found that working time affects gender specialization of households, which in turn affects fertility; Krafft [68] found that a reduction in female employment opportunities may be one of the drivers of rising fertility in Egypt. Therefore, the increase in employment opportunities may also reduce the fertility willingness of rural floating population. In sum, this study found that displacement significantly reduced farmers’ fertility intentions. This finding provides a reference for the Chinese government and other countries with low fertility rates for efforts to improve them.

“Live and work in peace and contentment” should represent the final destination of those in the floating population. For the rural floating population in particular, a suitable living environment and stable working environment may help to change their negative attitudes and emotions, unburden them from the conditions in which they are living and thereby greatly improve their willingness to bear children; this would not only change their attitudes around bearing children but also improve their mental health. For example, Mencarini et al. [69] highlighted that life satisfaction is conducive to the improvement of fertility intention in countries or regions with low fertility rates. Begall and Mills [70] found that in the case of low breeding rate rates, higher work pressure can significantly reduce mothers’ fertility intention. Further, Barbos and Milovanska-Farrington [71] found that Switzerland’s system of paid maternity leave improved people’s willingness to bear children, especially those who already had two children. Unfortunately, however, in terms of the overall environments in which China’s rural floating population live, individuals in this population remain far from suitable and stable living and working conditions. The current study looked specifically at China’s rural floating population, which stemmed from research on the established relationship between the floating population and low fertility rates, as well as findings on the fertility intentions of this unique group. At present, academic research on the fertility willingness and fertility preferences of individuals in the rural floating population is still in its infancy, and few people care about the fertility willingness of this group. Therefore, this study helps to provide a reference point for governments in efforts to formulate appropriate and effective policies and measures while also focusing on the notion of living and working in peace and contentment in their implementation.

The current study contains several limitations. First, the relationship between RPM and fertility willingness changes with changes in the times. Therefore, it would benefit future studies to explore the relationship between these factors. Second, ideally, panel data sets can be developed to also investigate changes in the relationship between RPM and fertility intention as the times change. Finally, this study used rural China as a case in point. In recent years, the Chinese government has been committed to improving social security policies for its rural floating population. Future research should further examine whether the findings of this study can be generalized to other countries and regions with low fertility rates.

## 6. Conclusions and Implications

This study quantified the impacts of rural population mobility on fertility intention using data from a large sample of Chinese rural residents. The study found that after the correction of sample selection bias, rural population migration significantly reduced individuals’ willingness to bear children. The main findings are as follows.

First, for rural residents who choose to move, if they chose not to move, their willingness to give birth would increase by 19.820%, their willingness to have female children would increase by 48.526% and their willingness to have male children would drop by 26.711%.

Second, for rural residents who choose not to move, if they chose to move, their willingness to give birth would drop by 55.982%, their willingness to have female children would drop by 18.294% and their willingness to have male children would drop by 55.106%.

Third, for eastern rural residents who choose to move, if they chose not to move, their willingness to give birth would decrease by 40.273%. For midwest rural residents who choose to move, if they chose not to move, their willingness to give birth would increase by 24.786%.

Fourth, for eastern rural residents who choose not to move, if they chose to move, their willingness to give birth would increase by 11.032%. For midwest rural residents who choose not to move, if they chose to move, their willingness to give birth would drop by 71.744%.

The findings of this study also point to several policy implications. First, improving the living and working conditions of those in the rural floating population is extremely important. Accordingly, improvements to the social security system and the stability of those in this population are vital. For example, we should establish a unified national social security system that integrates urban and rural areas, as well as form a unified social security system for employment, housing, medical care and aged care services. At the same time, since the workplaces of rural floating population are cities, local governments should increase the birth rates by improving the child-rearing environments in cities. For example, parental leave can be simplified by changing national laws or by companies taking unique measures, giving farmers and other workers enough personal time and space to increase their willingness to have children. In brief, the ultimate goal of people choosing to live and work is to live and work in peace and contentment, and living and working in peace and contentment can increase their willingness to bear children.

## Figures and Tables

**Table 1:** The definitions and descriptions of the variables in the model.

Variables	Definition	Mean	S.D.
**Dependent variable**			
FFI	The number of children the interviewee wanted to have (num)	1.930	0.649
DHG	The number of girls the interviewee wants to have (num)	0.964	0.483
DHB	The number of boys the interviewee wants to have (num)	0.972	0.440
**key variable**			
RPM	1 if interviewee’s household registration is outside the county (district, city) or another township, 0 otherwise	0.366	0.482
**Control variables**			
Gender	1 if interviewee is female, 0 otherwise	0.555	0.497
Age	The age of interviewee (years)	32.087	7.369
Education	0 if interviewee has a junior high school degree or below, 1 if interviewee has a high school degree, 2 if interviewee has a college degree, 3 if interviewee has a bachelor degree, 4 if interviewee has a graduate degree	0.599	0.946
Nationality	1 if the ethnicity of the interviewee is Han, 0 otherwise	0.916	0.277
Belief	1 if the interviewee has religious beliefs, 0 otherwise	0.091	0.288
Marriage	1 if the interviewee is married and cohabiting, 0 otherwise	0.790	0.407
Party	1 if the interviewee is in the Communist Party, 0 otherwise	0.033	0.180
Health	1 if the interviewee considers himself or herself to be in good health or relatively good health, 0 otherwise	0.704	0.457
Pension	1 if the interviewee has a pension, 0 otherwise	0.535	0.499
Fixed assets	The number of houses owned by the interviewee (num)	1.020	0.643
Lnincome	The logarithm of the interviewee’s annual household income (RMB)	10.609	1.508
Num-children	The interviewee’s number of biological children (num)	1.307	0.929
**Instrumental variable**			
IV	1 if interviewee agrees or relatively agrees with the statement that it is difficult for rural people to obtain urban household registration, 0 otherwise	0.461	0.499

**Table 2:** The collinearity test results.

Variables	Num-Children	Age	Marriage	Education	Lnincome	RPM	Pension
**VIF**	1.84	1.77	1.75	1.64	1.13	1.11	1.11
**Variables**	Health	Belief	Nationality	Party	IV	Fixed assets	Gender
**VIF**	1.10	1.10	1.09	1.06	1.04	1.03	1.03

**Table 3:** The mean differences in the variables between farmers who choose to move and those who do not.

Variables	Population Immobility		Population Mobility		Diff.	
FFI	1.956	(0.625)	1.883	(0.685)	0.073	**
DHG	0.973	(0.474)	0.950	(0.497)	0.023	
DHB	0.989	(0.424)	0.942	(0.465)	0.047	**
Gender	0.555	(0.497)	0.554	(0.498)	0.002	
Age	32.866	(7.325)	30.733	(7.252)	2.133	***
Education	0.432	(0.818)	0.890	(1.074)	−0.458	***
Nationality	0.901	(0.299)	0.943	(0.232)	−0.042	***
Belief	0.103	(0.304)	0.071	(0.257)	0.032	**
Marriage	0.815	(0.389)	0.748	(0.435)	0.067	***
Party	0.032	(0.176)	0.036	(0.187)	−0.004	
Health	0.675	(0.469)	0.754	(0.431)	−0.079	***
Pension	0.566	(0.496)	0.481	(0.500)	0.085	***
Fixed assets	1.065	(0.605)	0.943	(0.697)	0.121	***
Lnincome	10.398	(1.422)	10.976	(1.582)	−0.578	***
Num-children	1.412	(0.925)	1.125	(0.907)	0.287	***
IV	0.504	(0.500)	0.388	(0.488)	0.116	***
Observation	1100		634			

Note: Standard deviations are in parentheses; ** *p* < 0.05, *** *p* < 0.01.

**Table 4:** The estimates for the determinants of RPM and FFI.

Variables	Selection			FFI					
				Population Mobility			Population Immobility		
Gender	0.055	(0.83)		−0.105	(−1.84)	*	−0.061	(−1.68)	*
Age	0.047	(1.04)		0.036	(0.94)		−0.040	(−1.59)	
Age^2^	−0.001	(−1.00)		−0.001	(−0.98)		0.001	(1.36)	
Education	0.191	(4.35)	***	0.156	(4.47)	***	0.034	(1.11)	
Nationality	0.009	(0.07)		0.149	(1.20)		0.041	(0.65)	
Belief	−0.150	(−1.20)		0.122	(1.08)		0.181	(2.96)	***
Marriage	0.070	(0.62)		−0.034	(−0.36)		−0.181	(−2.89)	***
Party	−0.473	(−2.44)	**	−0.377	(−2.39)	**	0.107	(1.01)	
Health	0.022	(0.28)		0.033	(0.49)		−0.052	(−1.30)	
Pension	−0.330	(−4.68)	***	−0.199	(−3.23)	***	−0.077	(−1.82)	*
Fixed assets	−0.369	(−6.27)	***	−0.105	(−2.23)	**	0.008	(0.24)	
Lnincome	−0.386	(−5.73)	***	−0.140	(−2.35)	**	−0.040	(−0.91)	
Lnincome^2^	0.033	(7.51)	***	0.013	(3.17)	***	0.004	(1.44)	
Num-children	−0.045	(−0.92)		0.289	(6.50)	***	0.311	(12.52)	***
IV	−0.200	(−3.39)	***	-	-	-	-	-	-
Area dummies	YES			YES			YES		
Constant	−0.011	(−0.01)		0.526	(0.83)		2.527	(5.82)	***
lns1				−0.235	(−4.58)	***			
lns2				−0.529	(−13.02)	***			
r1				1.008	(8.44)	***			
r2				0.379	(1.75)	*			
Wald-chi2 (16)	120.43 ***								
LR test of indep. eqns.	17.61 ***								
Log likelihood	−2506.2152								
Observations	1734								

Note: *t*-values are in parentheses; * *p* < 0.10, ** *p* < 0.50, *** *p* < 0.01.

**Table 5:** The impacts of RPM on FFI.

Groups	Factual FFI	Counterfactual FFI	ATT/ATU	*t*-Value	Change (%)
Population mobility	1.887 (0.265)	2.261 (0.242)	−0.374 (0.141)	−26.256 ***	19.820
Population immobility	1.956 (0.256)	0.861 (0.275)	−1.095 (0.145)	−96.633 ***	−55.982

Note: Standard deviations are in parentheses; *** *p* < 0.01.

**Table 6:** The estimates for the determinants of RPM and DHG.

Variables	Selection			DHG					
				Population Mobility			Population Immobility		
Gender	0.085	1.26		−0.007	(−0.19)		0.025	(0.84)	
Age	0.030	0.65		0.034	(1.27)		−0.033	(−1.64)	
Age^2^	−0.000	−0.61		−0.001	(−1.44)		0.000	(1.52)	
Education	0.185	4.22	***	0.060	(2.22)	**	0.069	(3.07)	***
Nationality	0.095	0.70		0.048	(0.56)		0.038	(0.74)	
Belief	−0.153	−1.23		0.141	(1.77)	*	0.060	(1.22)	
Marriage	0.102	0.90		−0.106	(−1.70)	*	−0.041	(−0.82)	
Party	−0.411	−2.12	**	−0.133	(−1.20)		0.012	(0.14)	
Health	0.013	0.17		0.023	(0.50)		0.009	(0.28)	
Pension	−0.320	−4.52	***	−0.057	(−1.12)		−0.069	(−2.16)	**
Fixed assets	−0.387	−6.54	***	0.017	(0.37)		0.013	(0.53)	
Lnincome	−0.413	−6.13	***	−0.012	(−0.22)		−0.075	(−2.31)	**
Lnincome^2^	0.036	8.02	***	0.003	(0.67)		0.006	(2.84)	***
Num-children	−0.058	−1.18		0.169	(5.42)	***	0.194	(9.66)	***
IV	−0.119	−1.76	*	-	-		-	-	-
Area dummies	YES			YES			YES		
Constant	0.171	(0.22)		0.024	(0.05)		1.531	(4.46)	***
lns1				−0.735	(−16.71)	***			
lns2				−0.719	(−18.86)	***			
r1				0.179	(0.58)				
r2				0.677	(4.91)	***			
Wald-chi2 (16)	60.04 ***								
LR test of indep. eqns.	7.70 ***								
Log likelihood	−2057.0900								
Observations	1750								

Note: *t*-values are in parentheses; * *p* < 0.10, ** *p* < 0.50, *** *p* < 0.01.

**Table 7:** The estimates for the determinants of RPM and DHB.

Variables	Selection			DHB					
				Population Mobility			Population Immobility		
Gender	0.071	(1.05)		−0.111	(−2.90)	***	−0.078	(−2.97)	***
Age	0.041	(0.90)		−0.007	(−0.28)		−0.009	(−0.51)	
Age^2^	−0.001	(−0.85)		0.000	(0.43)		0.000	(0.29)	
Education	0.188	(4.24)	***	0.077	(3.24)	***	−0.038	(−1.77)	*
Nationality	0.078	(0.58)		0.072	(0.86)		−0.007	(−0.16)	
Belief	−0.145	(−1.17)		0.012	(0.15)		0.126	(2.87)	***
Marriage	0.067	(0.58)		0.057	(0.93)		−0.122	(−2.72)	***
Party	−0.386	(−2.01)	**	−0.215	(−2.04)	**	0.104	(1.38)	
Health	0.039	(0.51)		0.015	(0.33)		−0.067	(−2.31)	**
Pension	−0.363	(−5.14)	***	−0.107	(−2.49)	**	0.006	(0.19)	
Fixed assets	−0.365	(−6.11)	***	−0.080	(−2.39)	**	0.005	(0.22)	
Lnincome	−0.382	(−5.64)	***	−0.073	(−1.70)	*	0.047	(1.48)	
Lnincome^2^	0.033	(7.40)	***	0.006	(1.89)	*	−0.003	(−1.20)	
Num-children	−0.043	(−0.86)		0.121	(3.99)	***	0.115	(6.48)	***
IV	−0.147	(−2.15)	**	-			-		
Area dummies	YES			YES			YES		
Constant	0.001	(0.01)		0.744	(1.76)	*	0.944	(3.05)	***
lns1				−0.689	−11.17	***			
lns2				−0.862	−21.52	***			
r1				0.694	4.08	***			
r2				−0.382	−1.81	*			
Wald-chi2 (16)	65.03 ***								
LR test of indep. eqns.	4.06 **								
Log likelihood	−1919.4527								
Observations	1750								

Note: *t*-values are in parentheses; * *p* < 0.10, ** *p* < 0.50, *** *p* < 0.01.

**Table 8:** The impacts of RPM on DHG/DHB.

Groups	Factual DHG	Counterfactual DHG	ATT/ATU	*t*-Value	Change (%)
Population mobility	0.950 (0.147)	1.411 (0.165)	−0.462 (0.105)	−52.559 ***	48.526
Population immobility	0.973 (0.164)	0.795 (0.156)	−0.178 (0.091)	−26.020 ***	−18.294
Groups	Factual DHB	Counterfactual DHB	ATT/ATU	*t*-Value	Change (%)
Population mobility	0.943 (0.142)	0.727 (0.122)	0.216 (0.121)	30.965 ***	−26.711
Population immobility	0.989 (0.111)	0.444 (0.140)	−0.545 (0.111)	−1.0 × 10^2^ ***	−55.106

Note: Standard deviations are in parentheses; *** *p* < 0.01.

**Table 9:** The empirical results at the level of region.

Area	Groups	Factual FFI	Counterfactual FFI	ATT/ATU	*t*-Value	Change (%)
East	Population mobility	1.902 (0.242)	1.136 (0.327)	0.765 (0.290)	34.594 ***	−40.273
	Population immobility	1.976 (0.325)	2.194 (0.249)	0.218 (0.227)	8.781 ***	11.032
Midwest	Population mobility	1.868 (0.313)	2.331 (0.263)	−0.463 (0.163)	−19.488 ***	24.786
	Population immobility	1.950 (0.249)	0.551 (0.351)	−1.400 (0.233)	−93.554 ***	−71.744

Note: Standard deviations are in parentheses; *** *p* < 0.01.

## Data Availability

Data from the Chinese General Social Survey (CGSS) conducted by the China Survey and Data Center of Renmin University of China were used in this study. (http://cgss.ruc.edu.cn/, accessed on 12 May 2021).
